# Current Advances in Mathematical Modeling of Anti-Cancer Drug Penetration into Tumor Tissues

**DOI:** 10.3389/fonc.2013.00278

**Published:** 2013-11-18

**Authors:** MunJu Kim, Robert J. Gillies, Katarzyna A. Rejniak

**Affiliations:** ^1^Integrated Mathematical Oncology, H. Lee Moffitt Cancer Center and Research Institute, Tampa, FL, USA; ^2^Department of Cancer Imaging and Metabolism, H. Lee Moffitt Cancer Center and Research Institute, Tampa, FL, USA; ^3^Department of Oncologic Sciences, College of Medicine, University of South Florida, Tampa, FL, USA

**Keywords:** drug penetration, drug distribution, drug pharmacodynamics, tumor microenvironment, solid tumor, mathematical modeling

## Abstract

Delivery of anti-cancer drugs to tumor tissues, including their interstitial transport and cellular uptake, is a complex process involving various biochemical, mechanical, and biophysical factors. Mathematical modeling provides a means through which to understand this complexity better, as well as to examine interactions between contributing components in a systematic way via computational simulations and quantitative analyses. In this review, we present the current state of mathematical modeling approaches that address phenomena related to drug delivery. We describe how various types of models were used to predict spatio-temporal distributions of drugs within the tumor tissue, to simulate different ways to overcome barriers to drug transport, or to optimize treatment schedules. Finally, we discuss how integration of mathematical modeling with experimental or clinical data can provide better tools to understand the drug delivery process, in particular to examine the specific tissue- or compound-related factors that limit drug penetration through tumors. Such tools will be important in designing new chemotherapy targets and optimal treatment strategies, as well as in developing non-invasive diagnosis to monitor treatment response and detect tumor recurrence.

## Introduction

Systemic chemotherapy is one of the most widely used treatments in all kinds of cancers and at every stage of tumor progression. However, success of the systemic treatment depends not only on the efficacy of chemical compounds, but also on whether these compounds can reach all tumor cells in concentrations sufficient to exert therapeutic effect. Most clinically used anti-cancer drugs, however, lead to the emergence of anti-drug resistance, and to overcome this therapeutic limitation, the chemotherapeutic agents are often used in combination with other drugs of different pharmacokinetic properties or in combination with other anti-cancer treatments.

The process of drug delivery is complex and embraces different temporal and spatial scales, including the organism level (where drug absorption, distribution, metabolism, excretion, and toxicity are studied in various organs and are known together under the acronym ADME-T), tissue and cell scales (where the main processes include drug extravasation into the tumor tissue, its penetration via interstitial transport, and cellular uptake), and intracellular level (where drug internalization, intracellular pharmacokinetics, accumulation, and efflux are investigated). In this review, we will focus on these mathematical models that act on the tissue scale. We refer the reader to the following research papers and review articles that address the other modeling scales (1–11).

Transport of drug particles at the tissue level encounters several physiological and physical barriers. The architecture of tumor vasculature is leaky and tortuous when compared to the vasculature of normal tissues. As a result, the blood flow is chaotic and the supply of nutrients and drugs irregular. This, in turn, leads to the emergence of regions of transient or permanent hypoxia. The cellular and stromal architecture of tumor tissue is far from being as well organized as that of normal tissues, and it is characterized by increased cell packing density, high variability in tumor cell sizes, and their locations. Together, these result in a non-uniform exposure of tumor cells to metabolites and drugs. Elevated interstitial fluid pressure (IFP), which is a consequence of the lack of functional lymphatic vessels, and vascular hyperpermeability, reduce extravasation of both fluid, and drug molecules from the vascular system, hindering advective transport through the tumor tissue. A dense extracellular matrix (ECM) with irregular alignment of ECM fibers and with increased fiber cross-linking, also hinders the diffusion process. In general, it is difficult to predict the extent of drug penetration into the tumor tissue and to determine the influence of various microenvironmental factors on drug interstitial transport. The former issue can be addressed by developing imaging techniques to visualize either the drug uptake or its lethal effects. The latter can be tested using systematical computational simulations of properly formulated mathematical models.

Several imaging approaches have been used to visualize the effects of drug penetration into the tumor tissue, including naturally fluorescent drugs showing their spatial distribution (12–14), specific imaging biomarkers showing the effects of anti-cancer drugs, such as cell DNA damage (15, 16), intravital microscopic imaging for real-time *in vivo* drug distribution (17), or molecular photoacoustic tomography (18). Numerous imaging techniques and their use in oncology have been reviewed in Weissleder and Pittet (19), Gillies et al. (20), and Morse and Gillies (21).

Mathematical modeling provides tools for examining which of the various biophysical features of the tumor tissue and/or stroma and biochemical properties of drug compounds contribute significantly to limited drug penetration. *In silico* simulations are well-suited for testing combinations of multiple parameters that can be varied simultaneously in a controlled manner and over a wide range of values. Such a broad screening of drug or tissue conditions is rarely possible in laboratory experiments, but it is relatively easy and cheap in computer simulations. These theoretical screenings can help to determine the properties of therapeutic compounds optimal for their efficient interstitial transport (designing *in silico* drugs) or make decisions regarding the most effective drug combinations and scheduling protocols (designing *in silico* trials). Moreover, mathematical modeling allows for bridging laboratory experiments with clinical applications by providing the means to extrapolate the *in vivo* results from mouse models to humans. Recently, several review papers discussing the power of mathematical and biophysical modeling have been published (22–29).

In this review, we will focus on the most recent research articles that use mathematical and computational models of anti-cancer drugs acting on the cell and tissue scales. In the most general description, changes in the amount of drug present in the tissue depend on three values: the amount of drug entering the tissue (drug production), how the drug moves within the tissue (drug transport), and the amount leaving the tissue (drug elimination). However, various phenomena can contribute to each of these three processes. For example, a drug can be supplied from the preexisting vascular system or can be released within the tissue from a moving drug carrier (such as a nanoparticle), or it can be activated due to specific environmental conditions (for example, low oxygen level or high acidity). Drugs can be carried through the tissue with the interstitial fluid flow (advective transport) or move randomly due to the Brownian motion of drug molecules (diffusive transport). Drug elimination from the tissue can take place due to its natural half-life (decay), binding to the ECM (degradation or deactivation), or cellular uptake. Mathematically the simplest equation describing the kinetics of drug concentration *c*(*x,t*) at location *x* and at time *t* may be written as follows:

$$\underset{\text{change in drug concentration}}{\underset{︸}{\frac{\partial c(x,t)}{\partial t}}}=\underset{\text{DRUG PRODUCTION}}{\underset{︸}{\underset{\text{supply, release, activation}}{\underset{︸}{\kappa {|}_{\text{at region}}}}}}+\underset{\text{DRUG TRANSPORT}}{\underset{︸}{\underset{\text{diffusion}}{\underset{︸}{D\Delta c(x,t)}}-\underset{\text{advection}}{\underset{︸}{u(x,t)\cdot \nabla c(x,t)}}}}-\underset{\text{DRUG ELIMINATION}}{\underset{︸}{\underset{\text{decay, deactivation}}{\underset{︸}{\alpha c(x,t)}}-\underset{\text{cellular uptake}}{\underset{︸}{\beta c(x,t){|}_{\text{at cell}}}}}}$$

**Figure 1:**
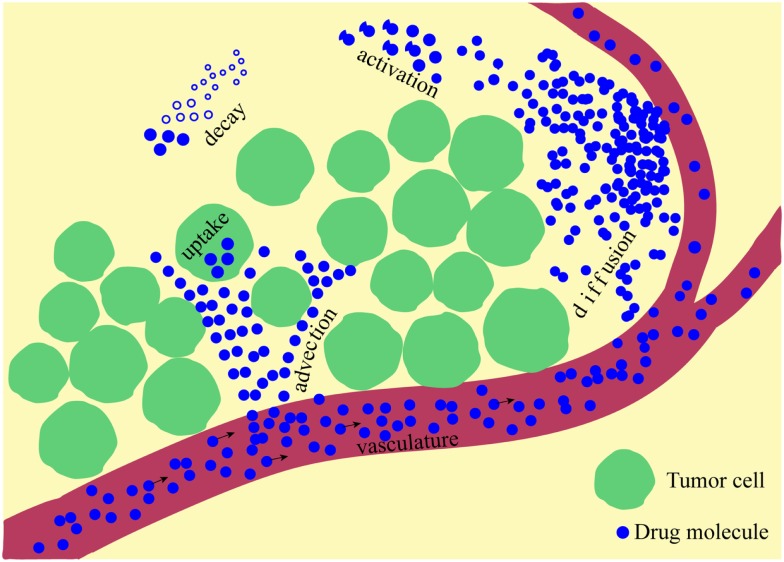
**A schematic representation of multiple physical processes involved in drug penetration into the tumor tissue**. Drug molecules are supplied from the vasculature and move through the interstitial space via diffusive and advective transports, can be activated and are subject to natural decay before they are taken up by the cells.

Here, κ is a constant rate of drug supply, release, or activation that takes place in a part of the domain (region), which may be a blood vessel (supply), nanoparticle (release), or low oxygen area (activation); *D* is a constant diffusion coefficient; *u*(*x,t*) is the velocity of the interstitial fluid; α is a decay or deactivation rate constant; and β is a rate constant of drug uptake by the cell. Schematically, all processes involved in the drug kinetics are shown in Figure 1. Notably, each of these factors may take a more complex form. A more detailed discussion regarding these processes follows below, and we give examples of how they have been addressed in the mathematical modeling literature and applied to anti-cancer drug kinetics.

## Models Addressing Drug Vascular Supply

**Figure 2:**
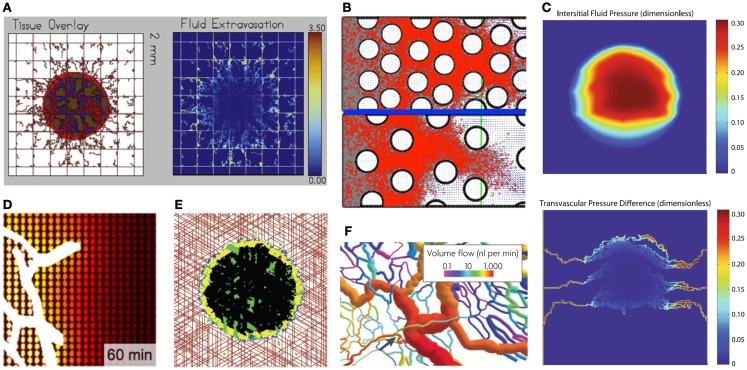
**Examples of typical outcomes from various mathematical models of drug penetration through the tumor tissue**. **(A)** A hybrid model of tumor mass and discrete vasculature (left, red-tumor tissue, brown-vasculature) was used to investigate fluid and drug extravasation from the vasculature (right, color corresponds to drug concentration) [from Wu et al. (37), Figure 18]; **(B)** Patterns of diffusion-(top) and advection-(bottom) dominated transport of drug molecules (red dots) through the interstitial space between the cells (white circles) [from Rejniak et al. (58), Figure 6]; **(C)** A gradient of the interstitial fluid pressure (top) and transvascular pressure differences (bottom) in the growing tumor mass [from Stylianopoulos et al. (30), Figure 2]; **(D)** A drug concentration gradient from the vessel (white) outward with colors representing high (yellow) and low (brown) levels of diffusive drug [from Thurber et al. (17), Figure 8]; **(E)** Spatial distributions of hypoxic (yellow), necrotic (black), and apoptotic (green) tumor cells within the tumor mass treated with angiogenesis inhibitors for 37 weeks; the treatment is supplied from vasculature (red) [from Gevertz (34), Fig.3]; **(F)** Structural adaptation of vessel diameters (colors represent the volume of blood flow) inside the tumors [from Pries et al. (31), Figure 6]. All figures reprinted with permissions.

After intravenous infusion, drug molecules circulate in the vascular system before they extravasate into the surrounding tissue. The drug influx rate κ is assumed constant in the equation listed above; however, more complex cases can be modeled wherein the vascular supply process depends not only on the molecule’s size, but also on the physical properties of the vasculature and the target tissue. In general, small drug or metabolite molecules can cross the vascular wall more easily than larger molecules can, and they can extravasate into both healthy and tumorous tissues. Larger molecules, such as nanoparticles, require vascular fenestration with larger pores to be able to leave the blood circulation system. Additional factors, such as electrostatic interactions between the particles and the negatively charged pores of the vessel wall, have been studied by Stylianopoulos et al. (30). The mathematical model predictions suggested that electrostatic repulsion has a minor effect on the transvascular transport of nanoparticles, but electrostatic attraction, caused even by small cationic charges, can lead to a significant increase in the transvascular flux of nanoparticles into the tumor interstitial space (Figure 2C).

The blood microcirculation within solid tumors is dysfunctional due to highly irregular vasculature (Figure 2F) that hinders delivery of both nutrients and drugs (31, 32). To investigate the distribution processes of small molecule drugs to cancer cells, a computational model based on fluorescent images of tumor functional vasculature was designed by Thurber et al. (17). The model was calibrated with experimental data and used to predict temporal changes in drug distribution profile around vessels with intermittent blood flow for a typical drug administration schedule (Figure 2D). Vascular images were also used by Baish et al. (33) to design a mathematical model that analyses drug diffusion in irregularly shaped domains based on two simple measures of vascular geometry. These include the maximum distance in the tissue from the nearest blood vessel and a measure of the shape of the spaces between vessels. This model can also predict how new therapeutic agents that inhibit or stimulate vascular growth alter the functional efficiency of the vasculature within the tumor tissue. Computational simulations of vasculature-targeting agents and their influence on tumor growth have been also performed by Gevertz (34, 35). These biophysical models (Figure 2E) were used to explore the therapeutic effectiveness of two drugs that target the tumor vasculature, angiogenesis inhibitors (such as *avastin*) and vascular disrupting agents (such as *combretastatin*). The simulation results suggested that vasculature-targeting agents, as currently administered, cannot lead to cancer eradication, although a highly efficacious agent may lead to long-term cancer control. The models, however, identified a treatment regimen that can successfully halt simulated tumor growth, even after the cessation of therapy.

Another computational study has been performed to test the effects that different drugs exert on the same mass of tumor tissue. Sinek et al. (36) compared the effectiveness of *doxorubicin* and *cisplatin* in vascularized tumors taking into account vascular and morphological heterogeneity. The simulation results showed that lesion-scale drug and nutrient distribution may significantly impact therapeutic efficacy. It has been also shown how the therapeutic effectiveness of *doxorubicin* penetration depends upon other determinants affecting drug distribution, such as cellular efflux and density, offering some insight into the conditions under which otherwise promising therapies may fail and, more importantly, when they will succeed. These simulations indicated that macroscopic environmental conditions, notably drug and nutrient distributions, give rise to considerable variation in lesion response, hence clinical resistance. Moreover, the synergy or antagonism of combined therapeutic strategies depends heavily upon this environment.

The elevated IFP and high hydraulic conductivity can act like microenvironmental barriers for transvascular transport to both anti-cancer drugs and nutrients, as have been investigated by Wu et al. (37). It has been shown computationally that small blood vessel resistance and collapse may contribute to lower transcapillary flux of oxygen. Moreover, the higher IFP distribution in the simulated tumors affected oxygen extravasation negatively, which, in turn, hindered tumor growth by decreasing the oxygen transfer to the tissue (Figure 2A). In another study Pozrikidis (38) has investigated the overall hydrodynamics of the leakage problem through a permeable capillary, taking into account hydraulic conductivity of arterial, venous, and extravasation flow rates. This showed that interstitium dilation promoted the rate of extravasation.

## Models Addressing Drug Release and Activation

To increase efficacy of therapeutic compounds and increase the time of drug survival inside the tumor tissue beyond its half-life, various methods of drug release and activation have been proposed. In our simple equation listed above, the release/activation rate κ is defined as a constant, and the release/activation region is hypothetical. However, more complex mechanisms can be incorporated in the models. The release region can represent a nanoparticle and can be varied in both space and time according to the changes in carrier locations; the activation rate may depend on local drug concentration or distribution of metabolites and may take place in hypoxic/acidic tumor areas, respectively, or may be stimulated by external factors, such as temperature or magnetic fields.

Nanoparticles have gained much interest as potential carriers of therapeutic agents due to their size, which enable them to extravasate in the leaky tumor vasculature preferentially, and due to their modular functionality, which allows for release of the drug by controlled diffusion from the core across the polymeric membrane to the matrix. A mathematical model taking into consideration avascular tumor growth followed by angiogenesis and nanoparticle-based drug delivery has been applied by van de Ven et al. (39) to design optimal therapeutic protocols. In particular, the effects of nanoparticles carrying *doxorubicin* were simulated for various parameter values to determine how much drug per particle and how many particles need to be released within the vasculature to achieve remission of the tumor. Moreover, it has been shown that cell death on a population level is non-linear with respect to the drug concentration. The same team has simulated vascular accumulation of blood-borne nanoparticles to analyze how nanoparticle vascular affinity depends on its size and ligand density, as well as vascular receptor expression (40). It has been shown that for high vascular affinities, nanoparticles tend to accumulate mostly at the inlet tumor vessels, leaving the inner and outer vasculature depleted of nanoparticles. For low vascular affinities, nanoparticles distribute quite uniformly in the intratumoral vasculature, but they exhibit low accumulation doses. It has been shown that an optimal vascular affinity can be identified by providing the proper balance between accumulation dose and uniform spatial distribution of the nanoparticles. This balance depends on the stage of tumor development (vascularity and endothelial receptor expression) and the nanoparticle properties (size, ligand density, and ligand-receptor molecular affinity). The timing and the location of drug release from nanoparticles have been investigated by Kim et al. (41) in a combination of *in vitro* experiments and mathematical modeling. It has been shown that gold nanoparticles carrying either *fluorescein* or *doxorubicin* molecules move and localize differently in an *in vitro* three-dimensional (3D) model of tumor tissue, depending on whether the nanoparticles are positively or negatively charged. Fluorescence microscopy and mathematical modeling show that uptake, not diffusion, is the dominant mechanism in particle delivery. These results indicate that positive particles may be more effective for drug delivery because they are taken up to a greater extent by proliferating cells. Negative particles, which diffuse more quickly, may perform better when delivering drugs deep into tissues.

Another drug carrier, engineered macrophages, that are capable of delivering pro-drugs to hypoxic areas within the tumor have been modeled by Webb et al. (42) and Owen et al. (43). In the former paper, two modes of action in the multicellular spheroids were investigated: either the macrophages delivered an enzyme that activated an externally applied pro-drug (bystander model), or they delivered cytotoxic factors directly (local model). The bystander model was comparable to traditional chemotherapy, with poor targeting of tumor cells in the center of the spheroid that are assumed hypoxic; on the other hand, the local model was more selective for the hypoxic regions. This work suggested that effective targeting of hypoxic tumor cells may require the use of drugs with limited mobility or whose action does not depend on cell proliferation. The latter article addressed a case where therapeutic macrophages were preloaded with nanomagnets and a magnetic field was applied to the tumor site. Both the conventional chemotherapy and chemotherapy with macrophages delivering hypoxia-inducible drugs were compared, and model simulations predicted that combining conventional and macrophage-based therapies would be synergistic, producing greater antitumor effects than the additive effects of each form of therapy. The model also revealed that timing is crucial in this combined approach with efficacy being greatest when the macrophage-based, hypoxia-targeted therapy is administered shortly before or concurrently with chemotherapy.

The effects of applying heat to tumors treated with *cisplatin* have been investigated by El-Kareh and Secomb (44). A theoretical model for the intraperitoneal delivery of *cisplatin* and heat to tumor metastases in tissues adjacent to the peritoneal cavity has shown increased cell uptake of drug, increased cell kill at a given level of intracellular drug, and decreased microvascular density. The model suggested that the experimental finding of elevated intracellular *platinum* levels up to a distance of 5 mm when the drug is delivered by a heated infusion solution is due to penetration of heat, which causes increased cell uptake of the drug. The effects of hyperthermia on chemotherapy were also investigated by Gasselhuber et al. (45) by developing a spatio-temporal model of the release of *doxorubicin* from low temperature sensitive liposomes. This model showed that this treatment combined with thermal ablation allowed for localized drug delivery with higher concentrations in the tumor tissue than conventional chemotherapy.

## Models Addressing Drug Diffusive Transport

In the model equation listed above, we used a constant diffusion rate *D* that leads to homogeneous diffusive transport. However, the diffusion may depend on the structure and other physical properties of the tissue in which this process occurs. One extension of the above equation has been widely used in modeling the spread of gliomas in the brain where the diffusion in the white matter and gray matter was characterized by different diffusion coefficients (46, 47).

In the context of drug penetration into the tumor tissue, Venkatasubramanian et al. (48) have created a mathematical model integrating intracellular metabolism, nutrient and drug diffusion, cell-cycle progression, and drug pharmacokinetics. Results indicated the existence of an optimum drug diffusion coefficient. A low diffusivity prevents effective penetration before the drug is cleared from the blood, and a high diffusivity limits drug retention. This result suggests that increasing the molecular weight of the anti-cancer drug by nanoparticle conjugation would improve its efficacy. The simulations also showed that tumors that grow fast are less responsive to therapy than are tumors growing more slowly with greater numbers of quiescent cells, demonstrating the competing effects of regrowth and cytotoxicity.

The complex interactions of drug particles and the ECM fibers that may hinder the drug molecule diffusion process have been modeled by Stylianopoulos et al. (49, 50). In this 3D model, stochastic fiber networks with varying degrees of alignment were considered. Quantitative analysis of four different structures, ranging from nearly isotropic to perfectly aligned, were performed. The results indicated that the overall diffusion coefficient is not affected by the orientation of the network. However, structural anisotropy results in diffusion anisotropy, which becomes more significant with an increase in the degree of alignment, the size of the diffusing particles, and the fiber volume fraction. These model predictions were validated experimentally, showing for the first time in tumors that the structure and orientation of collagen fibers in the extracellular space leads to diffusion anisotropy. The authors also investigated the effects of charge on the diffusive transport of macromolecules and nanoparticles in the ECM, taking into account steric, hydrodynamic, and electrostatic interactions. The model showed that electrostatic forces between the fibers and the particles result in slowed diffusion. However, the repulsive forces become less important as the fiber diameter increases. These results suggest that optimal particles for delivery to tumors should be initially cationic to target the tumor vessels and then change to neutral charge after exiting the blood vessels.

Since the ECM is composed of multiple cross-linked fibers, the drug particle diffusion in the interstitial space may rather resemble random movement through small nanochannels than diffusion through the open homogeneous space. A computational model that accounts for interface effects on diffusivity has been developed and validated by predicting experimental glucose diffusion through a nanofluidic membrane (51–53). Moreover, the passive transport of nanoparticles from bulk into a nanochannel has been modeled, showing that subtle changes in nanochannel dimensions may alter the energy barrier. This results in different nanoparticle penetration depths and diffusion mechanisms.

More detailed models of ECM structure, including fiber orientation, cross-link, and remodeling by the embedded cells have been developed by Bauer et al. (54, 55), Dallon and Sherratt (56) and Dallon et al. (57) in the context of vessel sprout and wound healing, respectively. These models have not yet been applied to model the role of ECM structure on drug molecule penetration. However, cellular heterogeneity of the stroma and its influence on both diffusive and advective forms of transport have been modeled by our group using idealized tissue morphologies of various porosity and cellularity values (58). Our simulations revealed that irregularities in the cell spatial configurations can solely result in the formation of interstitial corridors that are followed by drug or imaging agent molecules, leading to the emergence of tissue zones with less exposure to the drugs. Moreover, we showed that the relation between tissue porosity (defined as the extent of void space in the tissue), cellular density (defined as the number of cells per tissue area), and permeability (defined as time needed for a certain number of particles to traverse a predefined distance) is non-linear; thus it is also non-intuitive.

## Models Addressing Drug Advective Transport

During advective transport, drug molecules are carried with the flow of the interstitial fluid. This flow can arise from pressure differences within the tissue or from drainage of the fluid into the lymphatic circulation system. Wu et al. (37) investigated the role of the IFP, interstitial fluid flow, and the lymphatic drainage system on the transport of metabolites in developing tumors. The model simulations showed that elevated interstitial hydraulic conductivity combined with poor lymphatic function is the root cause of the development of plateau profiles of the IFP in the tumor, which have been observed in experiments.

At the macroscopic scale, where the individual cells are modeled as surrounded by the ECM space that is interpenetrated by the interstitial fluid, our group investigated the role of both advection and diffusion of drug molecules movement through the stroma (58). Simulation results collected from more than 100 different tissue morphologies showed that tissue cellular porosity and density influence the depth of drug penetration in a non-linear fashion. It has also been shown that for small diffusion coefficients, drug transport is advection dominated independently of tissue structure. Similarly, for all tissue structures considered in our simulations, drug molecule transport was diffusion dominated for large diffusion coefficients. However, for the intermediate values of fluid flow velocity and diffusion coefficients, the nature of interstitial transport depends strongly on the tissue morphology (Figure 2B). This indicates that sole knowledge of drug and tumor biophysical properties without knowledge of tumor tissue histology may lead to false predictions regarding the extent of drug penetration into the tumor tissue.

The significant role of the advective fluid flow in brain tumors has been investigated by Arifin et al. (59, 60). In this work, a computational model was employed to simulate 3D patient-specific distribution of *carmustine*. This model showed that a quasi-steady transport process is established within 1 day following treatment, and the drug is eliminated rapidly by transcapillary exchange, while its penetration into the tumor is mainly by diffusion. Convection appears to be crucial in influencing the drug distribution in the tumor resulting in non-homogeneous exposure to the drug: the remnant tumor near the ventricle is, by one to two orders of magnitude, less exposed to the drug than is the distal remnant tumor. In addition, local convective flow within the cavity appears to be a crucial factor in distributing the drug so that the tumor domain near the ventricle is prone to minimal drug exposure. The authors also simulated four chemotherapeutic agents (*carmustine, paclitaxel, fluorouracil*, and *methotrexate*) in a realistic 3D tissue geometry extracted from magnetic resonance images of a brain tumor. The simulation analysis showed that only *paclitaxel* exhibited minimal degradation within the cavity, as well as the best penetration of the remnant tumor.

A mixture of computational modeling and laboratory experiments on gels and tumors reported in Ramanujan et al. (61) showed that the diffusive transport of drug particles might be obstructed more significantly by collagen fiber alignment than particle movement due to fluid advection.

## Models Addressing Drug Decay, Deactivation, and Cellular Uptake

In our simple equation above, the rate of drug decay, deactivation, and cellular uptake were defined as proportional to the local drug concentration. In the case of drug decay this is a typical way of incorporating drug half-life. In the case of drug deactivation or degradation, these processes may also depend on environmental factors, such as binding to ECM fibers or interacting with other mocroenvironmental factors. This aspect of drug pharmacodynamics has usually been neglected in mathematical models due to insufficient experimental data to inform or validate the models. However, with the recent advances in visualizing and experimentally quantifying ECM fibril structure, this process should be easier to incorporate in future mathematical models. Additionally, the process of cellular uptake can depend on various factors. Certain drug molecules may bind to specific cell membrane receptors, and the efficacy of this process will then depend on the number of available receptors. Others may diffuse through the cell membrane, and this diffusion process will depend on both extracellular and intracellular drug concentrations.

The complex interplay between molecular size, affinity, and tumor uptake has been investigated by Schmidt and Wittrup (62) using a mechanistic model that takes into account drug molecular radius, interstitial diffusivity, available volume fraction, and plasma clearance. This model allowed for predicting the magnitude, specificity, time dependence, and affinity dependence of tumor uptake across a broad size spectrum of therapeutic agents. The authors concluded that the intermediate-size targeting agents (∼25 kDa) have the lowest levels of tumor uptake, when compared to tumor uptake levels achieved by smaller and larger agents. In Thurber and Wittrup (63), this model was extended to create a mechanistic description of total antibody uptake in a tumor, taking into account both free (unbound) antibody in the interstitium and antibody bound to its target. This allowed for an estimation of the time course of antibody uptake in solid tumors and its clearance from the blood plasma.

The cellular pharmacodynamics of various anti-cancer drugs was investigated by a mathematical model that takes into account cellular uptake of the drug and both intracellular and extracellular cytotoxicities. In El-Kareh and Secomb (64), the damage induced by *doxorubicin* was expressed as the sum of two terms, representing the peak values over time of intracellular and extracellular drug concentrations. Drug uptake by cells was assumed to include both saturable and unsaturable components, which provided better fits to *in vitro* cytotoxicity data. Model simulations suggested also a mechanism for the emergence of plateaus in the dose–response curve at high concentrations and short exposure time, as observed experimentally in some cases. Similar models were used to investigate the pharmacodynamics of *cisplatin* (65) and *paclitaxel* (66).

## Toward Clinical Applications of Mathematical Models

Mathematical models can also provide the means to scale experimental results from animal to human body size and metabolism, and can be used to test various drug administration procedures and schedules (bolus injections, dose-dense therapies, continuous infusions, and adaptive therapies) in virtual human body. El-Kareh and Secomb (67) used mathematical modeling to determine the optimal mode of delivery for *doxorubicin* by comparing three intravenous administration methods: bolus injection, continuous infusion, and liposomal delivery. The model took into account the relatively slow rate and saturability of *doxorubicin* uptake by cells and predicted peak concentrations of drug attained in tumor cells, as well as peak concentration of free *doxorubicin* in blood plasma. The model simulations suggested that continuous infusion for optimal durations is superior to the other delivery methods. A similar model, but using the tumor cord geometry, was used by Eikenberry (68) to test *doxorubicin* dose optimization. Model simulations showed that extending drug infusion time up to 2 h and fractionating large doses are two strategies that may preserve or increase anti-tumor activity, as well as reduce cardiotoxicity, by decreasing peak plasma concentration. Traina et al. (69) used the Norton-Simon tumor volume growth kinetic model (70) to predict a tolerable dose of *capecitabine* (7 days treatment followed by a 7-day rest) for advanced-stage breast cancer patients and this prediction was confirmed in phase I study. Traina et al. (71) continued to use the Norton-Simon model to optimize chemotherapeutic dosages and schedules in mouse xenograft models. Similar mathematical models have been used to study dose-dense chemotherapies (72) and to evaluate both the limitations of current schedules in breast cancer treatment and therapeutic advantages of novel dose-dense chemotherapies (73). Gatenby et al. (74) examined a novel approach in which cancer therapy was adapted to the evolving temporal and spatial variability of the tumor microenvironment, cellular phenotypes, and therapy-induced perturbations instead of using a typical linear protocol of drug administration. The developed mathematical model suggested that if resistant populations are present before administration of therapy, the total elimination of the drug-sensitive subpopulation will lead to the faster growth of a drug-resistant population. As an alternative, the simulated treatment was continuously modulated to control the size of tumor cell population that resulted in prolonged survival. The authors went a step further and, actually, tested their predictions experimentally. In subsequent work, Silva et al. (3) parameterized the adaptive therapy model using *in vitro* experiments and showed that this treatment strategy delays tumor burden and increases time to progression in tumor models.

The computational models are also well-suited to simulate treatments based on patient-specific parameters and tissue characteristics leading to personalized medicine. Our group investigates interstitial transport of drug and imaging agents using digitized samples of patients’ tumor histology (58). Frieboes et al. (75) implemented a mathematical model of tumor drug-response that integrates simulations with biological data and includes the experimentally observed resistant phenotypes of individual cells. This integrative method could be used to predict resistance based on specific tumor properties, potentially improving treatment outcome. Kim et al. (76) uses a combination of micro- and macroscopic imaging data and computational modeling to investigate blood flow in the heterogeneous tumor tissues. Venkatasubramanian et al. (77) uses breast cancer patients’ DCE-MRI (dynamic contrast-enhanced magnetic resonance imaging) data to predict their responsiveness to therapeutic treatment. Their model simulations showed that transvascular transport was correlated with tumor aggressiveness because of the formation of new vessels, and that increased transport heterogeneity led to increased tumor growth and poor drug-response.

Clinically used imaging techniques will be crucial in integrating mathematical models with clinical data in order to make patient-specific predictions. For example, DCE-MRI technique allows for collecting time-activity curves with high spatial and temporal resolution following a bolus injection of a Gadolinium-containing contrast agent, CA. The resulting data can be analyzed to generate spatially explicit (2D and 3D) maps of flow, perfusion, extracellular/extravascular volume fraction and, in some cases, water permeability (78, 79). In general, the delivery and extravasation of CA is modeled with standard 2- or 3-compartment PK models (80) and, as mentioned above, these maps can be used to infer drug distribution in human tumors (77, 81). While many investigators use ROI (region of interest) analyses to derive a single perfusion value to describe a tumor, it is becoming increasingly appreciated that enhancement is heterogeneous and that quantitative descriptors of this heterogeneity improve the precision for diagnosis and monitoring of therapy response (82–86). We contend that perfusion heterogeneity is a key factor in the response of tumors to therapy, both in terms of drug delivery and in the establishment of specific habitats that select for cells with specific phenotypes and hence, therapy responses (87).

## Conclusion and Future Directions

In this review, we discussed various mathematical models that were used to address different aspects of drug penetration through tumor tissue. All major stages of the penetration process have been investigated computationally: flow to different regions of tumors via blood vessels, crossing the vessel wall by drug molecules, their penetration through the interstitial tumor space, and cellular uptake. Mathematical models are well-suited to address such complex phenomena since by their nature they are able to handle multiple variables with numerous parameters. It is relatively easy and inexpensive to simulate tumor growth and treatment *in silico* and to compare differences in simulation outcomes when such parameters are changed simultaneously and over a wide range of values. In fact, this area of mathematical research is dynamically expanding. Especially novel are models that account for spatial aspects of drug transport. Half of the papers described in this review and all of the images collected in Figure 2 come from manuscripts that were published in the last 3 years, showing that this field is highly active and productive.

Typically in mathematical models, the drugs are defined as concentrations, as we did in the equation above. This is motivated by the fact that the number of drug molecules considered in the model might be a couple of orders of magnitude larger than the number of cells that form the tissue. However, in this description, only average behavior of drug molecules is captured. When more detailed drug kinetics need to be considered, such as molecule binding to cell receptors, intracellular trafficking, or mechanisms of drug extravasation from a vessel, drugs may be modeled as collections of individual molecules and can be traced individually in the model. Several novel models of this kind have been recently developed. Among models discussed in this review, the work of Ziemys et al. (51, 52), Mahadevan et al. (53), Frieboes et al. (40), and Rejniak et al. (58) traces the behavior of individual drug molecules and their interactions with the cells and/or vessels. In the first two papers the authors also discuss how to scale between the description of the kinetics of individual drug molecules and more general description of drug concentration.

Similarly, the more classical modeling approaches consider tumors as large populations of cells and represent them as cell densities (25, 36, 37, 40, 59, 64, 67, 68, 71, 73, 74, 81). These models can handle multiple cell subpopulations, but the number of different cell types has to be defined *a priori*, and new subpopulations cannot be dynamically created during the simulation. However, under specific conditions cells can be moved from one subpopulation to another. The predefined cell types may include a specific phase of the cell-cycle (a population of cells in G1, G0, S, or G2 phase), a particular cell phenotype (a population of proliferating, quiescent, hypoxic, or necrotic cells), or a particular cell response to the treatment (a population of drug-resistant or drug-sensitive cells). The advantage of continuous models is that they can handle large populations of cells, but the significant disadvantage is that all cell properties in these models must be averaged, since no individual cells are considered. In view of the growing evidence of heterogeneity of tumor cells on the genetic, phenotypic, and drug-response levels, the averaged cell properties and the averaged cell responses to anti-cancer treatments may not be sufficient to make predictions for individual patients.

In contrast, in the individual-cell-based models (called also the single-cell-based models, or the agent-based models) each cell is represented as a separate entity that acts as an independent agent according to some predefined rules (cell phenotype), but cell behavior can also be modulated by interactions with other cells and with the immediate cell microenvironment (selection forces). In this class of models cells may differ from each other significantly (cells may have distinct phenotypes, independently regulated cell-cycles, different levels of receptors, or different accumulations of mutations). Several models discussed in this review are single-cell-based (34, 35, 43, 52, 53, 58). The main advantage of these models is their natural cellular heterogeneity that better represents tumor multicellular composition than the continuous models. It is, of course, possible to analyze results of individual-cell-based models on a cell population level (in terms of average values and standard deviations, distributions, or correlations), similarly as this is done with experimental measurements. Moreover, these analyses can be compared to results from continuous models. The inverse process, that is extracting detailed information on individual cells from continuous models, is impossible. The main disadvantage of agent-based models is in their limitations to handle large number of cells. Typically, this limit is in thousands of cells, but with constantly increasing speed of computers and with development of novel faster computational techniques (parallel, GPU, or cloud computing) the number of cells that the model can handle in a reasonable time may not be a limitation anymore.

It is worth noting that every mathematical model is by its nature a simplification of the biological system it is assumed to represent, so we do not expect that one model will incorporate all processes involved in drug penetration through the tumor tissue. And we also do not expect that the unified modeling framework addressing all aspects of drug transport through tumor tissue will emerge in the near future. *In silico* models need to be designed to investigate a specific research question similarly to how biological experiments focus on the selected aspects of tumor treatment and do not address in a single experiment all possible combinations of involved factors. Computational models should not be too complex to allow for quantitative analysis of the relative importance of all features and parameters included in the model. However, in contrast to experiments, model parameters (e.g., drug molecular mass or charge, timing and dosing of drugs, and their activation or uptake properties) can be varied over a wide range of values and can be changed simultaneously in a controlled way, giving investigators insight into a full spectrum of drug properties that lead to the desired (or undesired) effects. These model outcomes will then provide guidance for further laboratory experimentation, and both results, positive and negative, will be informative for biologists. The positive results will suggest the environmental conditions or drug concentrations that are worth pursuing experimentally; the negative results will advise the drug concentrations or their properties that do not lead to a desired effect and can be omitted, reducing experimental costs and time. In fact, close collaboration between mathematical modelers, biologists, and clinicians is crucial, in our opinion, for making progress in improving anti-cancer treatments.

In our opinion the computational models of tumor development and treatment that will be successfully applied in personalized medicine need to be single-cell-based to be able to account for differences between tumors in individual patients (inter-tumor heterogeneity) and between distinct regions within the same tumor tissue (intra-tumor heterogeneity). Such models will be able to address phenotypic, genetic, and drug-response heterogeneity observable in patient tumors. The future models need to be temporal to capture the dynamics of tumor growth, cell–cell interactions, and response to therapy. These models will allow for temporal analysis of model results in order to identify more effective drug administration schedules with potentially variable schedules and dosages that cannot be intuitively inferred from analyzing drug properties in laboratory experiments. The future models should also be spatially explicit and three-dimensional, since both cell growth dynamics and drug transport dynamics are significantly different between the one-, two-, and three-dimensional spaces. *In vivo* tumors have complex geometries, variable cellular densities, irregular vasculature that cannot be captures by simple non-spatial, population-based models. And over all the future models need to be quantitative, based on quantitative experimental data (to inform and parameterize the model), and producing quantitative results, that can be compared to experimental measurements or clinical data.

Given the complexity of processes taking place during tumor development and its treatment, as well as significant inter-patient and intra-tumor variability, the cross-disciplinary approaches that integrate data and methods from various scientific disciplines have a better chance to delineate the mechanisms of tumor resistance to treatment and the way to overcome drug delivery barriers. The mathematical models that are properly integrated with experimental data, such that both *in silico* models and laboratory experiments inform each other, can provide tools for interpreting data, evaluating the most important parameters for designing new experiments, and developing strategies to improve tumor treatment.

## Conflict of Interest Statement

The authors declare that the research was conducted in the absence of any commercial or financial relationships that could be construed as a potential conflict of interest.
